# Allergic diseases of the skin and drug allergies – 2017. Rapid desensitization for delayed reactions to chemotherapy and monoclonal antibodies

**DOI:** 10.1186/1939-4551-6-S1-P104

**Published:** 2013-04-23

**Authors:** Maria Aranzazu Vega Castro, Ana María Alonso, Juan María Beitia, Maria Belén Mateo, Remedios Cardenas

**Affiliations:** 1 Allergy Service, Hospital Universitario De Guadalajara, Guadalajara, Spain; 2 Allergy Servicce, Hospital General De Ciudad Real, Ciudad Real, Spain

## Background

Drug desensitization is the induction of temporary clinical unresponsiveness todrug antigens to which patients have presented severe hypersensitivity reactions (HSR). Rapid desensitization in patients suffering immediate hypersensitivity reactions with chemotherapeutic agents and monoclonal antibodies have been widely described and have shown to be successful. Non-immediate hypersensitivity reactions with other drugs have usually required desensitization with several days’ protocols to achieve total doses.

## Methods

Thirty-eight desensitization procedures were performed in 5 patients with a 12-13 step, 6-hour protocol. All patients had developed a delayed maculopapular rash with the use of chemotherapeutic and/or biological agents. Three patients were pretreated with corticosteroids, paracetamol and antihistamines before each desensitization procedure.

## Results

All the 38 desensitizations undertaken were successfully completed (25 with Temozolamide, 4 with Bendamustine, 4 with Rituximab and 5 with Infliximab). We observed HSR during 8 (21%) of desensitizations, including 5 inmediate exantema and 3 delayed local macular exantema. Two patients were treated with corticosteroids and anti-histamines after the desensitization protocol to avoid more delayed HSR.

## Conclusions

Rapid desensitization protocols are safe and effective in getting over delayed HSR to chemotherapeutic and monoclonal antibodies and allow patients with severe diseases to continue their treatment.

